# Attitudes toward depression among rural primary healthcare providers in hunan areas, China: a cross sectional study

**DOI:** 10.1186/s12909-023-04197-1

**Published:** 2023-04-10

**Authors:** Jianfei Xie, Min Liu, Siqing Ding, Zhuqing Zhong, Sainan Zeng, Aizhong Liu, Shiwen He, Jianda Zhou

**Affiliations:** 1 Nursing Department, The Third Xiangya Hospital, Central South University, 138 Tongzipo Road, Changsha, Hunan 410013 China; 2 Xiangya Nursing School, Central South University, Changsha, China; 3 Xiangya School of Public Health, Central South University, Changsha, China

**Keywords:** Depression, Rural healthcare, Psychological training healthcare

## Abstract

**Background:**

Mental health services are not sufficient for depression patients in rural areas of China, training in mental health knowledge for primary healthcare providers has been encouraged, but the effect of this encouragement has rarely been reported.

**Methods:**

A cross-sectional survey was conducted in primary healthcare facilities that sought to include all the primary healthcare providers (registered physicians and nurses) in two cities in Hunan province, China by administering questionnaires that covered depression symptoms, typical depression cases, and the Revised Depression Attitude Questionnaire.

**Results:**

In total, 315 primary healthcare providers agreed to participate in the study and finished the questionnaires, of which 12.1% had training in depression. In addition, 62.9% of the rural primary healthcare providers were able to recognize most general depression symptoms, and 8.3% were able to recognize all general depression symptoms. The primary healthcare providers in the survey held a neutral to slightly negative attitude towards depression as indicated by their professional confidence (mean scores 16.51 ± 4.30), therapeutic optimism/pessimism (mean scores 29.02 ± 5.98), and general perspective (mean scores 18.12 ± 3.12) scores. Fewer rural primary healthcare providers knew (28.3%) or applied (2.9%) psychological intervention in the clinic.

**Conclusions:**

Our study indicated that primary healthcare providers knew about general depression symptoms, but lacked psychological intervention skills and held low confidence in and pessimistic attitudes toward depression care. We therefore speculate that existing psychological training for primary healthcare providers is insufficient in quantity and quality, making the need to explore more effective types of training urgently.

## Background

Nowadays, amount of studies has focused on mental health. Mental health is one of the important elements in health and it includes physical and mental coordination, adaptation to the social environment, well-being, and bringing one’s ability into full play in work [1]. The mental health in rural areas in China is not optimistic: high prevalence of mental health problems. For rural preschoolers, 70% of them were classified as having at least one kind of mental health problem [2]. For rural children, 14.50% and 10.22% of them experienced loneliness and depression [3]. Children who are left behind in the care of other family members when their parents migrate to urban areas were reported to have serious mental health status 2.7 times more than non-left-behind children [4, 5]. For rural caregivers, they reported having a prevalence of depressive symptoms (32%), anxiety symptoms (42%), and stress symptoms (30%) [6]. For rural elderly, geriatric depression rates in the east, west, and central rural area were up to 24.34%, 30.11%, and 36.77%, respectively [7]. Rural empty-nest elderly also reported a significantly higher prevalence of depression (OR:1.55; 95%CI [1.03–2.35]) than not-empty-nest elderly [8]. Thus, there is great demand for mental health services in rural areas in China.

When rural people with depression face mental health problems, rural primary healthcare institutions are their first choice [9]. However, psychiatric services in China are provided primarily by municipal-level general hospitals, specialized hospitals, and psychiatric prevention institutions. Rural healthcare facilities do not typically have specialized psychiatric departments, rural residents had lower access to specialist doctors than urban residents [10, 11]. Most physicians who work in rural healthcare facilities are general practitioners, and although some of them may have had some training in psychology, these providers are unlikely to be specialized in mental health or neurology. Therefore, mental health services are limited in rural primary healthcare institutions.

For improving mental health services in rural primary healthcare institutions, integrating mental health treatment into primary care has been recognized as a global priority [12]. Prior studies have reported that rural primary healthcare providers possessed a good knowledge of mental health, with an approximately 87.7% of accuracy rate for mental health knowledge, and showed better abilities to acquire mental health knowledge and to recognize cases than healthcare providers who worked in urban communities [13, 14]. These studies were limited, however. Primary healthcare providers’ knowledge of mental health was reported but not psychological barriers such as depression. Any lack of guidance from which the providers may have suffered also was less reported. Therefore, primary healthcare providers’ ability to help prevent and treat depression in rural areas still lags behind the demand for such services.

With the imbalance between supply and demand for mental health services for depression patients in rural areas of China, training in mental health knowledge for primary healthcare providers has been encouraged, but the effect of this encouragement has rarely been reported. Consequently, the first aim of this study is to report the status of these providers’ psychological training. The second aim of this study is to report the status of rural Chinese primary healthcare providers’ abilities to recognize depression as well as their attitudes toward depression.

## Methods

### Study settings and participants

This cross-sectional study was carried out in Lianyuan and Leng Shuijiang cities in Hunan province, China, totaling 39 rural healthcare facilities. We obtained the rosters of rural healthcare facilities from the applicable bureaus of public health. Those rural primary healthcare providers included in the study were registered physicians and nurses who were clinical workers with at least one year of experience. Those excluded were managers, part-time workers, interns, and mentors or experts from municipal hospitals.

According to previous data, it is found that rural primary healthcare providers possessed an 82.08% of accuracy rate for mental health knowledge. We set the design efficiency as 2 and required that with 95% confidence, the results need to fall within 10% of the overall truth rate. According to the calculation formula N = Z^2^_1−α/2_(1-p)/ε^2^γ, it is calculated that N = 84. Considering the 20% of attrition rate and the design efficiency, the questionnaires required for this cross-sectional study were 202 in total.

### Implementation

Rural primary healthcare providers were recruited between May 2014 and May 2018 from 39 rural healthcare facilities in Hunan province, China. To be eligible, the rural healthcare facilities agreed to participate and signed informed consent via e-mail. Five members of our study group went to the rural healthcare facilities and spent 3 days at each healthcare facility. Under the help of the president of rural healthcare facility, we called the medical staff to conference room at off-duty time, handed out questionnaires and consent forms to the primary healthcare providers and informed them of the purpose of this study. The providers were then asked to fill out the questionnaires within 30 min.

All the collected data were entered into Epi Data 3.0 software by two of our group’s members (postgraduate nursing students who have received data entry training) simultaneously and were saved into different data files. The data was confirmed by comparing the two sets of data. If any variable contains missing or incomplete data, the corresponding case will be excluded from the analysis.

### Questionnaires

Questionnaires consisted of the following five parts.

### Demographic questionnaire

A 6-item demographic questionnaire was applied to collect physicians’ and nurses’ gender, age, profession (physician or nurse), years of experience, educational background, and marital status.

### Depression symptoms questionnaire

The depression symptoms questionnaire was composed of eleven depression symptoms from the Classification and Diagnostic Criteria of Mental Disorders in China (CCMD-3). The CCMD-3 is a set of relevant diagnostic scales and has been published as a diagnostic guideline in China that has been widely used to rate mental health problems and diseases [15]. The range of Cronbach’s coefficient was 0.848–0.959 [16]. The eleven general depression symptoms were estimated by 11 single-choice questions, 1 point for each question, for 11 points for the total score of the depression symptoms questionnaire. The diagnostic standards of CCMD-3 and American Diagnostic and Statistical Manual of Mental Disorders (DSM-IV) are consistent in depression diagnosis, with a consistent rate of 0.997, a sensitivity of 0.919, a specificity of 0.999, a positive predictive value of 0.978, a negative prediction value of 0.997 and the KAPPA value is 0.946 [17].

The CCMD-3 defined the symptom standard of depressive episode, as mainly in low mood, and at least the following four symptoms: (1) Loss of interest, no pleasure; (2) Lack of energy or fatigue; (3) Psychomotor retardation or agitation; (4) Low self-evaluation, self-blame, or guilt; (5) Difficulty in association or decreased ability to think consciously; (6) Recurrent thoughts of death or suicide or self-injury; (7) Sleep disorders, such as insomnia, early awakening, or excessive sleep; (8) Loss of appetite or weight; (9) Hyposexuality. And it defined depression with psychotic features as in addition to the above standard symptoms, added with psychotic symptoms such as “Intermittent production of hallucinations “or” Discontinuous stiffness”. The depression symptoms questionnaire was composed of the above 11 symptoms.

### Typical case questionnaire

This questionnaire presented a typical depression case for evaluation that was taken from the Investigation and Evaluation Scheme of Mental Health Work Indicators [18]. The content was as follows: Mr. Li felt very sad, and tired, and had insomnia every night for the past several weeks. Additionally, he doesn’t want to eat and has lost weight. It has been hard for him to concentrate on his business, make decisions, and even to deal with household chores. When he visited the clinic many times for sleep problems many times, he remained silent.

### Revised depression attitude questionnaire (R-DAQ)

Depression Attitude Questionnaire (DAQ) had shown problems in psychometric properties and suitability for the health professionals. The R-DAQ is a revised tool for examining clinicians’ views toward and understanding of depression and has been found to have with good internal consistency (Cronbach’α = 0.84) and satisfactory test-retest reliability: intraclass correlation coefficient was 0.62 (95% C.I. 0.37 to 0.78) as well [19]. The Chinese version of this study showed Cronbach’α = 0.93.

The R-DAQ consists three parts: professional confidence, therapeutic optimism/pessimism and a generalist perspective. The section on professional confidence in depression care, which has 7 items, measures how confident a provider feels in giving professional depression care. The section on therapeutic optimism about depression, which has 10 items, measures if a provider can define depression and its treatment correctly. The generalist perspective section is about depression occurrence, recognition, and management and consists of 5 items. A 5-point Likert scale is used for the 22 items, and scores can range from 22 to 110. The response options are 1 = Strongly disagree, 2 = Disagree, 3 = Neither agree nor disagree, 4 = Agree, and 5 = Strongly agree. Lower scores indicate a more negative/pessimistic view of depression and its management.

### Clinical application questionnaire

The Clinical Application Questionnaire was composed of the three questions: “Do you use psychological scales to identify psychological symptoms, such as Self-Rating Depression Scale or Self-Rating Anxiety Scale?”; “Do you knowing psychological intervention methods, such as modified behavioral activation, mindfulness-based cognitive therapy or electroconvulsive therapy?”; and “Do you use psychological interventions method at work, such as modified behavioral activation, mindfulness-based cognitive therapy or electroconvulsive therapy?”.

### Statistical analysis

To analyze the data from our questionnaires, we used SSPS 19.0 software. Percentages, (X ± S) were used to describe the participants’ demographics, psychological trainings, depression symptoms recognition ability, and attitude toward depression. We statistically tested, the difference of depression symptoms recognition ability and attitude toward depression between participants with different demographics were analyzed by using a two-sample t-test or completely random design. And the difference of depression typical case recognition and clinical application of psychological knowledge between participants with different demographics were analyzed by using chi-square tests. Multivariate analysis were used to analyze the correlation of demographic information and depression symptoms, depression case recognition, and R-DAQ.

## Results

### Characteristics of participants

**Fig. 1:**
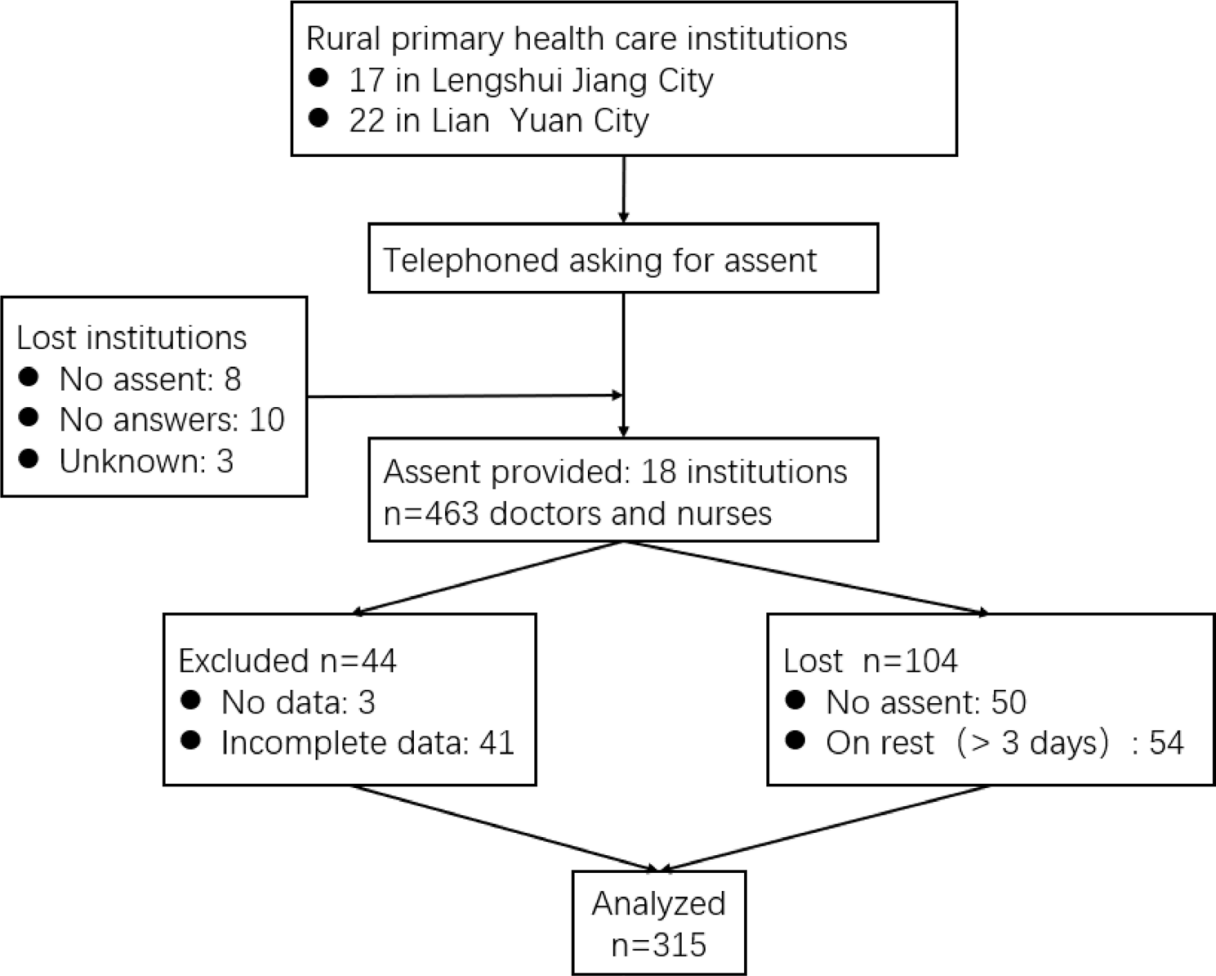
The flow of rural health-care facilities and health care providers

As reported in Fig. 1, eighteen rural healthcare facilities and 463 rural primary healthcare providers (physicians and nurses) were invited to participate in the study. Eighteen rural healthcare facilities and 315 rural primary healthcare providers signed the consent forms and finished questionnaires.

Across this study, most of the participants were physicians, were more than 30 years old, had over 10 years’ experience, and had less than a bachelor’s degree. The providers who had ever participated in psychological training were presented in Table 1.

**Table 1:** The demographic characters of primary health care providers in rural

characteristics of participants		N(%)	psychological trainings		X^2^	*P*
			Trained	Not trained		
Gender	Male	149(47.3)	54(67.5)	95(40.4)	17.55	0.001
	Female	166(52.7)	26(32.5)	140(59.6)		
Age (years old)	≤ 30	39(12.4)	6(7.5)	33(14.0)	36.64	0.001
	31 ~ 40	111(35.2)	9(11.3)	102(43.4)		
	≥ 41	165(52.4)	65(81.3)	100(42.6)		
Profession	Physicians	237(75.2)	74(92.5)	163(69.4)	17.15	0.001
	Nurse	78(24.8)	6(7.5)	72(30.6)		
Working years	≤ 10 years	62(19.7)	8(10.0)	54(23.0)	6.36	0.01
	≥ 11 years	256(80.3)	72(90.0)	181(77.0)		
Education background	Below Bachelor degree	268(85.1)	68(85.0)	200(85.1)	0.001	0.98
	Bachelor degree and above	47(14.9)	12(15.0)	35(14.9)		
Marital status	Married	281(89.2)	76(95.0)	205(87.2)	3.74	0.05
	Not married	34(10.8)	4(5.0)	30(12.8)		
Training topic	Depression	315(100)	38(12.1)	277(87.9)		

### Knowledge of general depression symptoms and the typical case

The mean score of knowledge in general symptoms of depression (total score 11) was 8.54 ± 1.70, 8.3% of the providers got full scores in general symptoms of depression, 62.9% of the providers were able to recognize more than 8 general symptoms of depression, and 83.8% of the providers were able to recognize depression correctly in the typical case example. As reported in Table 2, the symptoms “Intermittent production of hallucinations” and “Discontinuous stiffness” had the lowest correct responses, 44.4% and 43.2% respectively.

**Table 2:** The recognition of common symptoms and typical case of depression (n = 315)

Questions Which of the following belongs to general symptoms of depression?	Answer	
	Right(%)	Wrong(%)
1.Loss of interest, no pleasure(yes/no)	308(97.8)	7(2.2)
2.Intermittent production of hallucinations(yes/no)	140(44.4)	175(55.6)
3. Lack of energy or fatigue(yes/no)	272(86.3)	43(13.7)
4.Psychomotor retardation or agitation(yes/no)	243(77.1)	72(22.9)
5.Low self-evaluation, self-blame, or guilt(yes/no)	271(86.0)	44(54.0)
6.Difficulty in association or decreased ability to think consciously(yes/no)	225(76.4)	90(28.6)
7.Discontinuous stiffness(yes/no)	136(43.2)	179(56.8)
8.Recurrent thoughts of death or suicide or self-injury(yes/no)	291(92.4)	24(7.6)
9.Sleep disorders, such as insomnia, early awakening, or excessive sleep(yes/no)	287(91.1)	28(8.9)
10.Loss of appetite or weight(yes/no)	253(80.3)	62(19.7)
11. Hyposexuality(yes/no)	264(83.8)	51(16.2)
*Case recognition*: Mr Li almost feel very sad, tired and have insomnia every night in recent weeks. Besides, he didn’t want to eat and lost weight. It’s hard for him to concentrate on the business, make decisions and even deal with household chores. When he visited to clinic for sleep problems many times, he was silent.	264(83.8)	51(16.2)

### Attitudes to depression

Three parts of R-DAQ are expressed in Table 3. The providers in this study showed a neutral to slightly negative attitude towards depression (R-DAQ score), and providers who had received psychological training showed no significant difference in the R-DAQ compared to those who did not.

**Table 3:** Attitude to geriatric depression estimated by R-DAQ items (n = 315)

R-DAQ items	Agreement (%)	Trained		Not trained	
		Mean	SD	Mean	SD
**Professional confidence in depression care**		16.66	4.20	16.46	4.34
• I feel confident in assessing depression in patients	24(7.6)	2.20	0.82	2.13	0.90
• I feel comfortable in dealing with depressed patients’ needs	36(11.4)	2.49	0.81	2.48	0.83
• I feel confident in assessing suicide risk in patients presenting with depression	51(16.2)	2.58	0.99	2.59	0.96
• It is rewarding to spend time looking after depressed patients	119(37.8)	3.21	0.76	3.17	0.78
• I am more comfortable working with physical illness than with mental illnesses like depression (RS)	279(88.6)	2.41	0.74	2.47	0.78
• My profession is well placed to assist patients with depression	16(5.1)	1.86	0.81	1.82	0.85
• My profession is well trained to assist patients with depression	21(6.7)	1.91	0.87	1.80	0.91
**Therapeutic optimism/pessimism about depression**		29.63	5.78	28.82	6.04
• Psychological therapy tends to be unsuccessful with people who are depressed (RS) *	72(22.9)	4.45	0.78	4.09	0.90
• Depression reflects a response which is not amenable to change (RS)	128(40.6)	3.79	0.84	3.84	0.83
• Becoming depressed is a way that people with poor stamina deal with life difficulties (RS)	167(53.0)	3.36	1.00	3.21	0.98
• There is little to be offered to depressed patients who do not respond to initial treatments (RS)	312(99.0)	1.80	0.74	1.71	0.77
• One of the main causes of depression is a lack of self-discipline and will-power (RS)	313(99.4)	1.76	0.68	1.71	0.77
• Antidepressant therapy tends to be unsuccessful with people who are depressed (RS)	99(31.4)	4.04	0.88	3.88	0.96
• Becoming depressed is a natural part of adolescence (RS)	138(43.8)	3.59	1.03	3.48	1.15
• Depression treatments medicalise unhappiness (RS)	295(93.7)	1.79	0.84	1.80	0.89
• Once a person has made up their mind about taking their own life no one can stop them (RS)	298(94.6)	1.80	0.086	1.81	0.86
• Becoming depressed is a natural part of being old (RS)	210(66.7)	3.25	1.07	3.29	0.95
**Generalist perspective about depression occurrence, recognition and management**		18.19	3.27	18.10	3.07
• All health professionals should have skills in recognizing and managing depression	235(74.6)	4.16	1.02	4.00	1.00
• Recognizing and managing depression is often an important part of managing other health problems	130(41.3)	3.39	0.82	3.42	0.74
• Anyone can suffer from depression	112(35.6)	3.28	1.04	3.31	0.91
• People with depression have care needs similar to other medical conditions like diabetes, COPD or arthritis.	140(44.4)	3.56	0.95	3.54	0.87
• Depression is a disease like any other (e.g. asthma, diabetes)	191(60.6)	3.80	0.93	3.83	0.85

**RS**: reverse scoring

**Agreement**: combining agree and strongly agree

**SD**: standard deviation

*: *p* < 0.05

### Psychological knowledge in clinical application

**Fig. 2:**
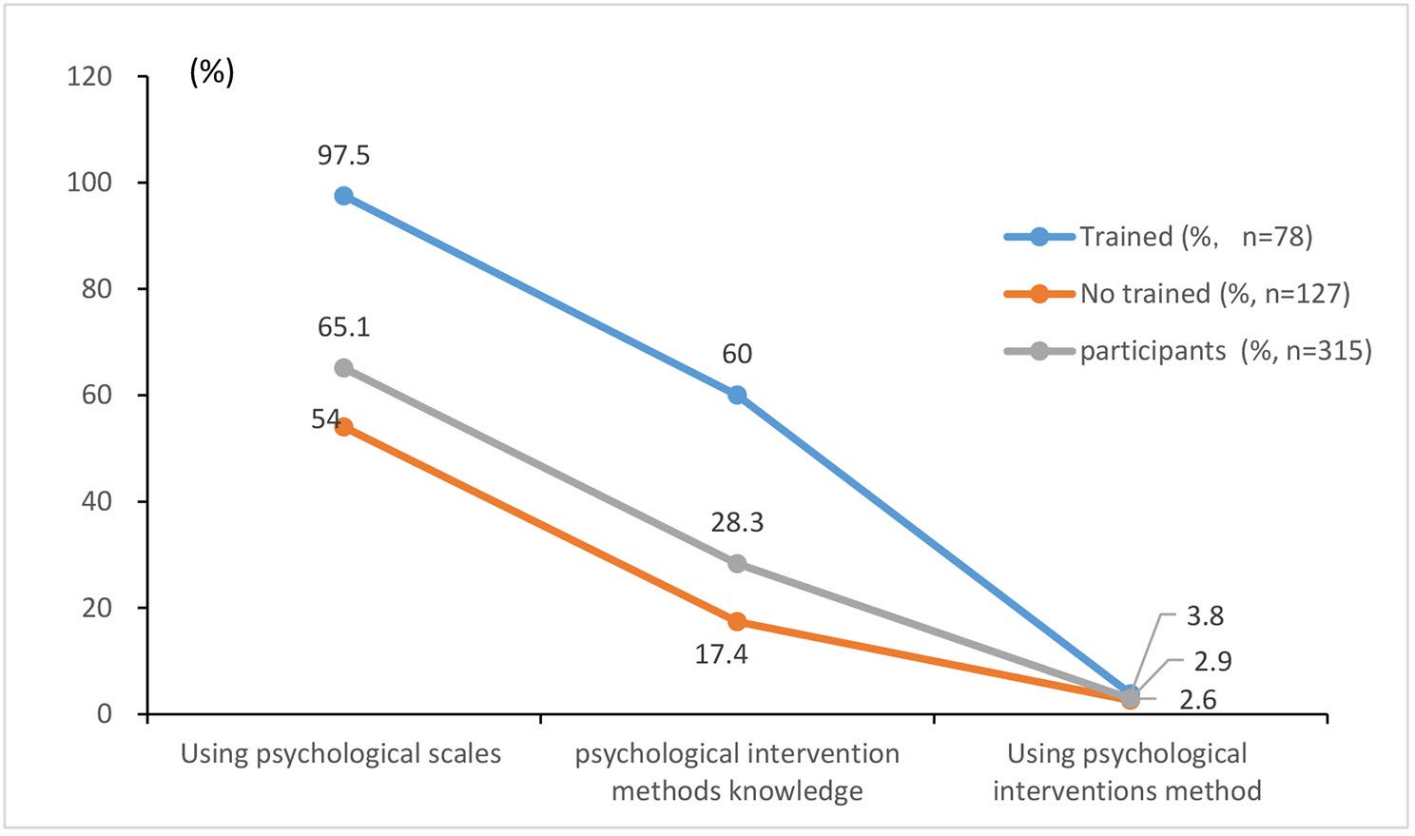
The clinical application of psychological knowledge

As reported in Fig. 2, providers who ever participated in psychological training showed better scores in the use of psychological scales (P＜0.001) and knowledge of psychological intervention methods (P＜0.001) but not in the use of psychological intervention methods (trained:3.8%, no trained:2.6%, P = 0.580).

### Factors impacting depression symptom knowledge, case recognition, and R-DAQ scores

The independent risk factors of depression symptom knowledge, case recognition and R-DAQ scores were showed in Table 4. There was no significant difference in depression symptom knowledge, case recognition and R-DAQ scores, between nurses and physicians. As reported in Table 5, education background showed positive correlation with depression symptom knowledge, case recognition, and R-DAQ scores (all p < 0.001). Psychological trainings was positively related to depression symptom knowledge, case recognition, and R-DAQ scores, but it had no statistically significance.

**Table 4:** Symptoms, case recognition and R-DAQ scores in different participants (n = 315)

characteristics of participants		Symptoms ‾(X ± S)	*P* value	Case recognition (%)		*P* value	R-DAQ ‾(X ± S)	*P* value
				right	wrong			
Profession	Physicians	8.44 ± 1.75	0.067	204(86.1)	33(13.9)	0.057	64.38 ± 12.40	0.067
	Nurse	8.85 ± 1.50		60(76.9)	18(23.1)		61.44 ± 11.92	
Gender	Male	8.41 ± 1.69	0.199	128(85.9)	21(14.1)	0.339	64.35 ± 12.90	0.344
	Female	8.66 ± 1.71		136(81.9)	30(18.1)		63.03 ± 11.80	
Age (years old)	≤ 30	9.05 ± 1.52	0.003	36(92.3)	3(7.7)	0.204	74.64 ± 13.65	< 0.001
	31 ~ 40	8.80 ± 1.57		89(80.2)	22(19.8)		65.05 ± 12.57	
	≥ 41	8.24 ± 1.78		139(84.2)	26(15.8)		60.12 ± 9.97	
Working years	≤ 10 years	8.98 ± 1.71	0.022	55(88.7)	7(11.3)	0.243	64.48 ± 14.05	0.555
	≥ 11 years	8.43 ± 1.69		209(82.6)	44(17.4)		63.45 ± 11.90	
Education background	Below Bachelor	8.26 ± 1.65	< 0.001	218(81.3)	50(18.7)	0.005	63.56 ± 12.41	0.747
	Bachelor and above	10.15 ± 0.93		46(97.9)	1(2.1)		64.19 ± 11.97	
Marital status	Married	8.50 ± 1.73	0.190	231(82.2)	50(17.8)	0.378	62.97 ± 12.16	0.005
	Not married	8.85 ± 1.42		30(88.2)	4(11.8)		69.29 ± 12.47	
Psychologic-al trainings	Yes	8.86 ± 1.63	0.049	75(93.8)	5(6.3)	0.005	64.47 ± 12.10	0.491
	No	8.43 ± 1.72		189(80.4)	46(19.6)		63.37 ± 12.42	

**R-DAQ**: Revised Depression Attitude Questionnaire

**Table 5:** Multivariate analysis of Symptoms, case recognition and R-DAQ scores in different participants

Characters		Coefficients	SD/OR	t/95%CI	P values
Symptoms	Constant	3.10	1.01	3.08	0.002
	Profession	0.12	0.25	0.47	0.64
	Gender	0.21	0.21	1.04	0.3
	Age	-0.10	0.22	-0.46	0.649
	Working years	0.24	0.14	1.70	0.089
	Education background	1.22	0.13	9.47	< 0.001
	Marital status	-0.03	0.11	-0.23	0.82
	Psychological trainings	0.34	0.20	1.67	0.097
Case recognition	Working years	0.27	1.31	0.92–1.87	0.129
	Education background	1.08	2.93	1.67–5.15	< 0.001
	Psychological trainings	0.81	2.25	0.94–5.34	0.067
	Constant	-3.23	0.04		0.014
R-DAQ	Constant	5.66	4.74	1.20	0.233
	Profession	0.41	1.16	0.36	0.721
	Gender	2.59	0.96	2.69	0.008
	Age	0.64	1.02	0.63	0.532
	Working years	-0.19	0.67	-0.29	0.773
	Education background	14.08	0.61	23.27	< 0.001
	Marital status	0.14	0.53	0.27	0.787
	Psychological trainings	0.63	0.96	0.66	0.511

**R-DAQ**: Revised Depression Attitude Questionnaire

## Discussion

Depression is a treatable mental health problem, and most depression patients recover if they receive early depression recognition and appropriate intervention [20]. This study indicated that psychological training had a good effect on depression symptom recognition, depression case recognition, clinical practice, and attitude towards depression, which were the same as prior studies [13, 21]. The views on mental illness for healthcare providers, especially nonpsychiatric healthcare providers, were related to personal experience and professional training [19, 22]. Training in mental healthcare could encourage primary healthcare providers’ clinical practices to become more focused on patients’ mental health [23]. When more rural primary healthcare providers were encouraged to participate in depression training, they demonstrated better mental health practice competency [24].

The existing psychological training for primary healthcare providers is insufficient in quantity and quality. Participation in psychological training in Hunan areas (25.4%) is lower than that in Cameroon (49.1%) [25]. Guangxi areas showed the same situation:13.3% of rural primary healthcare facilities had more than 20 healthcare providers participating in psychological training per year [13]. The quality of mental health work in rural areas is also lower than in urban areas [26]. Medical staves may have forgotten their lessons and were not well-experienced in handling depression [26]. Younger and more highly educated providers reported better knowledge grasping [21, 27]. This study showed no difference between physicians and nurses in the ability to depression symptoms and case recognition, even though physicians had a higher percentage of participating in psychological training. Only primary care physicians’ training in mental health is insufficient [27], nurses spend a lot of time with patients and are more likely to discover patients’ mental health problems. Psychological training for nurses also should be encouraged.

This study reported that most rural primary healthcare providers recognized depression symptoms and typical depression cases correctly. Prior studies [13, 24, 28] indicated that primary care physicians had good knowledge about depression and symptoms related to psychosis, especially in primary healthcare providers responsible for mental health prevention and control. 71.4% of rural primary healthcare providers considered mental healthcare and professional knowledge of mental health as their required competencies [29]. That implied that providers had realized the importance of recognizing and managing depression.

This study found that primary healthcare providers held a neutral to slightly negative attitude toward depression. Rural primary healthcare providers had less confidence and more pessimistic attitudes toward depression care [28, 29], especially those with below bachelor’s degrees and junior titles [21]. Primary care physicians with stigmatizing attitudes posed tremendous barriers to offering treatment. With higher levels of stigma, they were more likely to refer mental health patients to a psychiatrist [28]. Isabelle and colleagues [25] showed that about 66% of primary healthcare providers agreed that most cases of depression they encountered originated from recent misfortune and felt uncomfortable working with depressed patients. Detection, treatment, and management of mental illness in clinical practice affected healthcare providers’ attitudes toward mental health [24]. healthcare providers’ views about mental illness could affect their problem-identification abilities and treatment decisions [19]. Understanding primary healthcare providers’ experiences and views was good for designing effective training in rural or low-resource communities [30].

This study showed that primary healthcare providers’ knowledge and attitude toward depression were inadequate to solve psychological problems, such as recognizing and identifying patients with mental disorders, communicating with patients, and dealing with emergencies [31]. Primary healthcare providers had limited experience in pharmacogenetic testing and less knowledge of evidence base in psychiatric medications [32]. Only 1.8% and 15.2% of primary healthcare providers knew a standard tool used to diagnose depression or have ever prescribed psychotropic drugs, respectively [25]. Rural primary healthcare providers also were hesitant to discuss psychiatric diagnoses because of concerns about negatively impacting a patient’s reputation [33]. Therefore, it is urgent to improve providers’ ability to solve psychological problems in the clinic.

Insufficient clinical knowledge and experience were the main barriers to recognizing and treating psychological problems in clinics for healthcare providers [34]. A good external environment and psychological training could facilitate primary healthcare providers to solve psychological problems. Constructing a stable funding mechanism, sustainability control over an organization’s budgeting decisions, and stability in funding renewal were necessary [35]. Building efficient information systems for accessing psychotherapeutic medicines timely, collaborating with psychologists and psychiatrists, and sharing the experience on screening, diagnosis, and treatment of common mental disorders were effective [22, 26]. In medical education, long-term mentoring was widely applied and showed a good effect on independent medical tasks, and enabled mentors to provide guidance, resources, encouragement, constructive criticism, and support to their mentees [36]. A whole-course mentoring system, in which mentors summarize and solve the main problems during the standardized training, could improve self-efficacy and job satisfaction for graduated nurses [37]. Thus, applying the whole-course mentoring system in psychological training may help primary healthcare providers to familiarize themselves with clinical practice and deliver specialist mental healthcare fast. Moreover, increasing the number of training, setting targeted training on depression, encouraging the training for nurses, and establishing a positive attitude towards depression in training also should be paid much attention.

### Strengths and weaknesses of this study

This study is the first to our knowledge to apply the R-DAQ to investigate primary healthcare providers in rural China and to combine it with questions about knowledge of common depression symptoms and the recognition of a typical depression case. The study revealed the states of psychological training on the primary healthcare providers and assessed the primary healthcare providers’ knowledge, skills, and attitudes toward depression in particular. However, it still has some limitations. First, the attrition rate was high. After taking into account the 20% rate of loss to follow-up, the study sample size was set to at least 202 participants by calculation. Although finally, 32% of physicians and nurses dropped out for various different reasons, the study sample size (315 participants) was adequate. Two factors likely contributed to our high attrition. One is that most participants were over 30 years old and may have had low interest in this research. Another is that healthcare providers in rural China work on two or three shifts, so one-third or half of the participants were rested at home and not enrolled each day. It would effectively control the attrition rate if extending the investigation time on each primary healthcare institution.

## Conclusion

This study reported that existing psychological training for primary healthcare providers in rural China is insufficient in quantity and quality. Although rural Chinese primary healthcare providers could correctly recognize most general depression symptoms, they had low confidence in their abilities to deal with depression and pessimistic attitudes toward depression care. These findings would help improve the efficacy of training on mental health knowledge for primary healthcare providers. The training objectives would be not limited to doctors but also nurses, the number and content of the training would be more targeted, and the providers’ attitude towards depression should be focused during psychological training.

## Data Availability

The datasets used and/or analyzed during the current study are available from the corresponding author on reasonable request.
